# Prevalence of diagnosed temporomandibular disorders among Saudi Arabian children and adolescents

**DOI:** 10.1186/s10194-016-0642-9

**Published:** 2016-04-22

**Authors:** Amal Al-Khotani, Aron Naimi-Akbar, Emad Albadawi, Malin Ernberg, Britt Hedenberg-Magnusson, Nikolaos Christidis

**Affiliations:** Orofacial Pain and Jaw Function, Department of Dental Medicine, Karolinska Institutet, SE-Box 4064, SE-141 04 Huddinge, Sweden; Scandinavian Center for Orofacial Neurosciences (SCON), Huddinge, Sweden; Department of Oral and Maxillofacial Surgery, Karolinska University Hospital, Huddinge, Sweden; Jeddah Dental Speciality Center, Ministry of Health, Jeddah, Saudi Arabia; Department of Clinical Oral Physiology at the Eastman Institute, Stockholm Public Dental Health (Folktandvården SLL AB), SE-113 24 Stockholm, Sweden

**Keywords:** Temporomandibular disorders, Prevalence, Children, Adolescents, RDC/TMD

## Abstract

**Background:**

Studies have indicated that the prevalence of symptoms and signs of temporomandibular disorders (TMD) are rare early in childhood, but become more prevalent in adolescents and adulthood. To our knowledge, no study has investigated the prevalence of TMD-diagnoses in children in the general population. The aim was thus to investigate the prevalence of TMD-diagnoses among children and adolescents in the general population using the Research Diagnostic Criteria for TMD (RDC/TMD).

**Methods:**

The current cross-sectional study consisted of 456 children and adolescents, aged between 10 and 18, randomly enrolled from 10 boy’s- and 10 girl’s- schools in Jeddah. The participants first answered two validated questions about TMD-pain, followed by a clinical examination according to RDC/TMD.

**Results:**

One hundred twenty-four participants (27.2 %) were diagnosed with at least one TMD-diagnosis. Myofascial pain was the most common diagnosis (15 %) followed by disc displacement with reduction, arthralgia, myofascial pain with limited mouth opening and osteoarthrosis. Children diagnosed with myofascial pain more often reported orofacial pain, headache and tooth clenching (*p* < 0.05), whereas children with arthralgia more often reported orofacial pain and tooth grinding than those without a TMD-diagnosis (*p* < 0.05). Only 18 % of the subjects in the TMD group had sought a dentist or physician for their pain.

**Conclusion:**

TMD was common among children and adolescents in Saudi Arabia. Self-reported orofacial pain and headache as well as bruxism were associated with a TMD-pain diagnosis and disc displacement. A surprisingly low percentage of children and adolescents sought treatment by a dentist or physician for their pains.

## Background

Temporomandibular disorders (TMD) are the main non-dental cause of pain in the orofacial region among children and adolescents [1, 2]. TMD-pain involves not only the temporomandibular joint (TMJ) and the masticatory muscles, but may also spread to adjacent structures such as teeth, ears, neck, head and back muscles [3]. Studies have showed that the prevalence of TMD-symptoms and signs are rare early in childhood [4, 5], but become more prevalent in adolescents and adulthood [2, 6]. Furthermore, like other chronic pain conditions, TMD have serious consequences regarding patient’s daily life at many levels; physical incapability, impaired sleep quality, and reduced learning abilities as well as its impact on expenditures [7]. A study also reported that adequate care of an adolescent with chronic pain necessitates a lot of time, energy, and affection from the child’s parents [8].

In contrast to adults, earlier studies regarding the prevalence of TMD in children and adolescents are not based on TMD-diagnoses after clinical examinations but have focused on the prevalence of TMD-symptoms and signs [9, 10], associated risk factors [11], and self-reported TMD-pain [2]. Previous studies indicated that the prevalence of self-reported TMD-symptoms in children and adolescents in the general population range from 1 to 50 % [9, 10, 12], and TMD-pain from 1 to 22 % [2, 11, 13]. The prevalence of TMD-signs in children and adolescents range from 3 to 33 % [14, 15]. The considerable worldwide variations of TMD prevalence may be attributed to differences in methodology, examination procedure, population selection, and the country of origin. A review evaluating TMD assessments in children and adolescents reports that about half of the included studies had methodological shortcomings, suggesting that there is a need of more adequate methods for diagnosing TMD in children and adolescents [16].

Studies have used the Research Diagnostic Criteria for TMD (RDC/TMD) [17] in children and adolescents, showing good reliability [18]. These diagnostic criteria have been used as gold standard for both children and adults. However, the recently published Diagnostic Criteria for TMD (DC/TMD) [19], are not yet validated for children.

In order to increase the level of reliability between different studies the RDC/TMD criteria have been recommended [20–22]. However, the prevalence of TMD-diagnoses according to these criteria have been reported only among adults [20, 21] and adolescents [22]. To our knowledge, no studies have yet investigated the prevalence of TMD-diagnoses in children in the general population. Therefore, the aim of this study was to investigate the prevalence of TMD-diagnoses in children and adolescents according to RDC/TMD. Secondarily, background factors associated with TMD-diagnoses were also investigated.

## Methods

This epidemiological, cross sectional, and randomized study was carried out in the city of Jeddah, Saudi Arabia. It was approved by the local ethical committee at the Department of Medical Study and Research at the Ministry of Health, Jeddah, Saudi Arabia. The participants received written and oral information about the study and written consent was obtained from a parent to all participants, prior to inclusion. The study was followed the guidelines of the Declaration of Helsinki and the STROBE statement (STrengthening the Reporting of OBservational studies in Epidemiology) [23].

### Participants

**Fig. 1:**
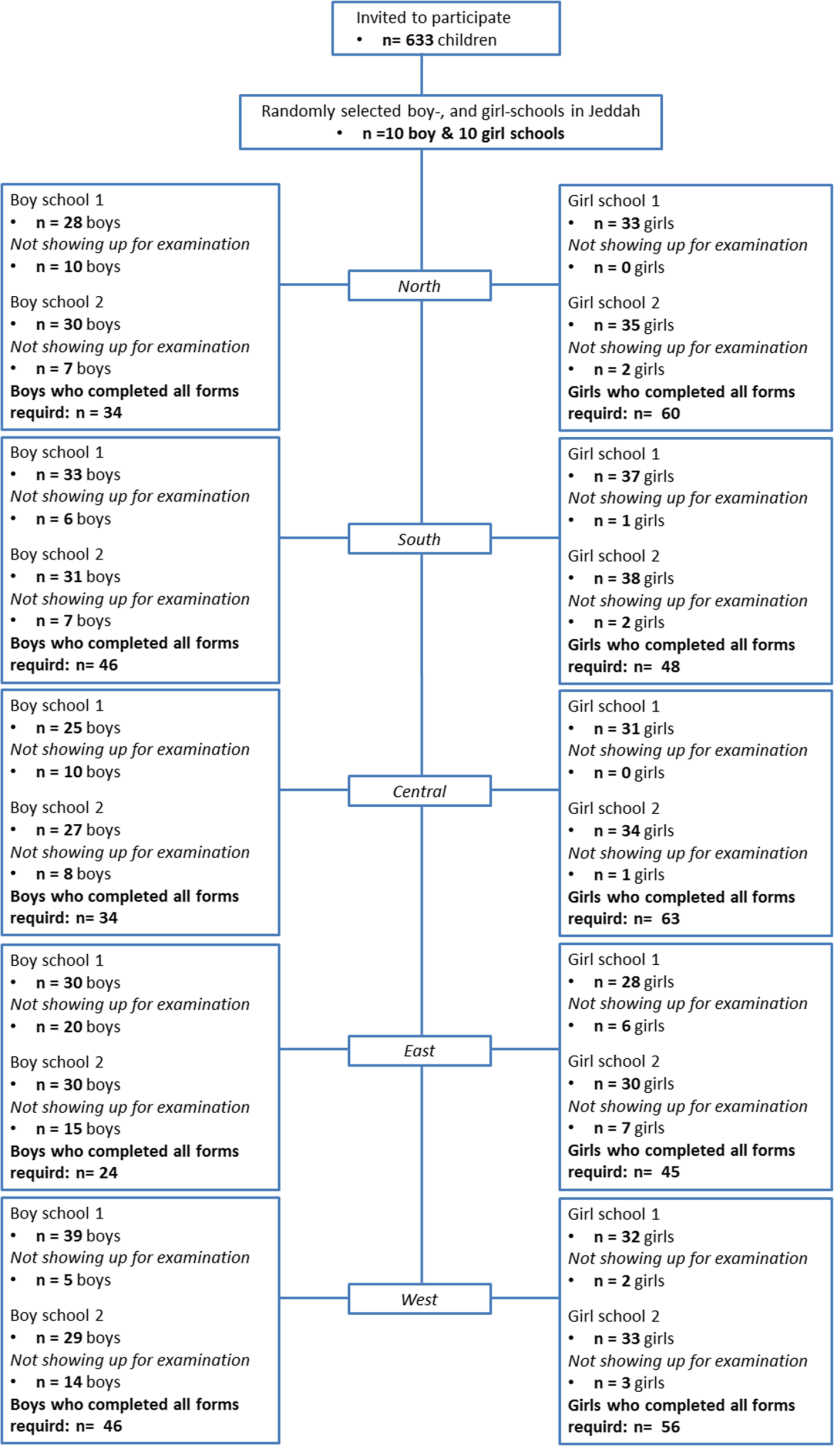
Flowchart of the participating children and adolescents. Flow-chart illustrates the inclusion of 456 boys and girls children among the general population in Jeddah, Saudi Arabia

The present study did not have any exclusion criteria, so all invited participants were included. This was done in order to achieve generalizable results. A total of 633 children and adolescents (the term children is used for both from now on) were invited to participate in order to have a sufficient number of participants, due to the risk of children not showing up for examination. According to the power calculation 450 children were necessary to detect true odds ratios for disease of 0.538 up to 1.860 with a power of 90 % and a significance level of *p* < 0.05. A total of 456 children completed the self-reported questionnaires and participated in the clinical examination were therefore included in the study (Fig. 1). They were between 10 and 18 years of age.

In order to obtain a varied sample, the present study followed the pre-defined set of school as clustered by the Ministry of Education. For that reason, Jeddah city was divided into the five regions; North, South, East, West, and Central. In addition, the education in Saudi Arabia is based on single-sex schools. Thus, from each region two schools with boys and two schools with girls were randomly selected, and from each school one class with an average of 30 students was randomly selected. The randomization was performed with an internet-based application (www.randomization.com) by a researcher (NC) who did not participate in data collection.

### Study design

Due to cultural considerations, there were two study designs, one for boys and one for girls. The main difference was that girls were examined in their school nurse’s room using a mobile dental chair, whereas the boys and their parents/guardians were invited to the dental clinic of the primary health care center in each region for examination.

#### Boys’ protocol

Before the clinical examination, proper information about the purpose of the study and a brief explanation of the questionnaires were presented to all children and their parents/guardians. The RDC/TMD history questionnaire (Dworkin and LeResche, 1992), in Arabic language, was then distributed [17]. In addition to the RDC/TMD questionnaire, questions regarding participant characteristics, medical history, presence of oral parafunctions, headache, previous trauma to the face, and use of oral appliances (occlusal appliances for TMD treatment and functional removable appliances for orthodontic treatment) were added. Further, data from the Jaw Disability Checklist (JDL) and the scores for the Graded Chronic Pain Scale (GCPS) included in the questionnaire were also retrieved.

Just before the examination, the examiner (AA-K), repeated the questions from the questionnaire to assure that the child had understood. The child was also asked additional questions about oral history including: a) oral parafunctions, such as daytime clenching and grinding, thumb sucking, nail/object biting, tongue thrusting and mouth breathing; b) previous orthodontic or TMD treatment; c) presence of headache; and d) trauma to the orofacial region.

Then, the boys were asked about the presence of orofacial pain using two validated questions (hereafter called S-TMD-pain), not included in the RDC/TMD, but commonly used to screen patients for TMD pain conditions [2, 18], *1) “Do you have pain in the temple, face, temporomandibular joint, or jaws once a week or more?” 2) “Do you have pain when you open your mouth wide or chew once a week or more?”*.

Finally, a clinical examination was performed for each participant. The clinical examination included both a brief intra-oral examination of dental and periodontal status, reporting presence or absence of caries and gingivitis, as well as an examination of the temporomandibular system according to the RDC/TMD protocol [17] by one examiner (AA-K). The examiner was trained in this procedure by an orofacial pain specialist with vast experience of the RDC/TMD examination (ME), and who has also been calibrated to a gold-standard examiner (Thomas List). In order to avoid any influence on the examination the accompanying parent/guardian was asked to wait outside the clinical room. If the parent/guardian insisted to attend, they were asked to remain passive during the entire session.

#### Girls’ protocol

In contrast to the boys, the information regarding the purpose of the study and the explanation of the questionnaires were distributed in sealed envelopes one day before the clinical examination to all girls. The rest of the protocol was exactly the same as for the boys.

### Data preparation and statistics

The participants were diagnosed using the RDC/TMD Axis I Diagnostic Algorithms [17], divided into three groups: Group I, Muscle Diagnoses (Myofascial pain and myofascial pain with limited opening); Group II, Disc Displacements (with and without reduction); Group III, Arthralgia, Arthrosis, Arthritis.

Descriptive statistics are presented as mean (SD) or median (interquartile range, IQR) depending on distribution of data, number of subjects and frequencies (%), as well as odds ratios (OR) with 95 % confidence interval (CI). In univariate analyses associations between TMD-diagnoses and potential predictors were performed using the Chi^2^-test. Differences in maximum voluntary mouth opening (MVO) between children with and without a TMD-diagnosis was analyzed with Student’s *t*-test. To analyze potential predictors to the different TMD-diagnoses in a multivariate model logistic regression was used to calculate OR. The regression model included the diagnosis as the dependent dichotomous variable, and sex (male/female), age (the children were clustered into two age-groups; 10–13 years/14–18 years), Saudi Arabian nationality (yes/no), grinding (yes/no) and clenching (yes/no) as independent dichotomous variables. Family income included 3 categories (below average/average/above average) based on the average income in Saudi Arabia for the year 2013 (15 000 SR/month) (www.cdsi.gov.sa) was modeled as a dichotomous dummy variable. Statistical analyses were performed using STATA 12 SE. 95 % CI not including 1 and p-values less than 0.05 were considered as statistically significant.

## Results

### Study population

Table 1 presents the demographic characteristics and medical as well as oral health for boys, and girls separately and for all children. No sex differences were found. Allergy was the most prevalent health condition (*n* = 57), followed by Asthma (*n* = 43), various anemia conditions (*n* = 14), bone diseases (*n* = 3), gastrointestinal tract disease (*n* = 3), diabetes (*n* = 2), heart disease (*n* = 2), Kidney disease (*n* = 2), hormonal disease (*n* = 1), autoimmune disease (juvenile idiopathic arthritis) (*n* = 1), and congenital syndromes (*n* = 1). There were no differences between TMD and non-TMD groups regarding demographic characteristics, or medical and oral health.

**Table 1:** Demographic data and general as well as oral health in 456 children/adolescents from a general population in Jeddah, Saudi Arabia. Figures show number of subjects (%) unless other is stated

	Boys	Girls	Total
	*n* = 184	*n* = 272	*n* = 456
Age			
Mean (SD)	14.8 (2.4)	13.5 (2.1)	14.0 (2.3)
10–13 years	69 (37.5)	166 (61)	235 (51.5)
14–18 years	115 (62.5)	106 (39)	221 (48.5)
Nationality			
Saudi Arabian	130 (70.7)	161 (59.2)	291 (63.8)
Non-Saudi Arabian^a^	54 (29.4)	111 (40.8)	165 (36.2)
School-level			
Primary (grade 1–6)	47 (25.5)	166 (61)	213 (46.7)
Intermediate (grade 7–9)	77 (41.9)	62 (22.8)	139 (30.5)
Secondary (grades 10–12)	60 (32.6)	44 (16.2)	104 (22.8)
Parental income			
Below average	78 (44.3)	155 (58.1)	233 (52.6)
Average	63 (35.8)	86 (32.2)	149 (33.6)
Above average	35 (19.9)	26 (9.7)	61 (13.8)
Living with			
Both parents	167 (92.8)	255 (93.8)	422 (93.4)
One parent	13 (7.2)	17 (6.3)	30 (6.6)
General health			
Allergy	24 (13)	33 (12.1)	57 (12.5)
Asthma	23 (12.5)	20 (7.4)	43 (9.4)
Other diseases^b^	8 (4.3)	21 (7.7)	29 (6.4)
Previous surgery	24 (13.5)	30 (11.2)	54 (11.8)
Oral health			
Caries	133 (72.3)	210 (77.5)	343 (75.4)
Gingivitis	60 (32.6)	75 (27.7)	135 (29.7)

^a^Middle East, Gulf Area and Africa

^b^ Heart disease, hormonal diseases, blood disease, congenital syndromes, bone disease, autoimmune diseases, gastrointestinal tract diseases and kidney

### TMD-diagnoses

**Fig. 2:**
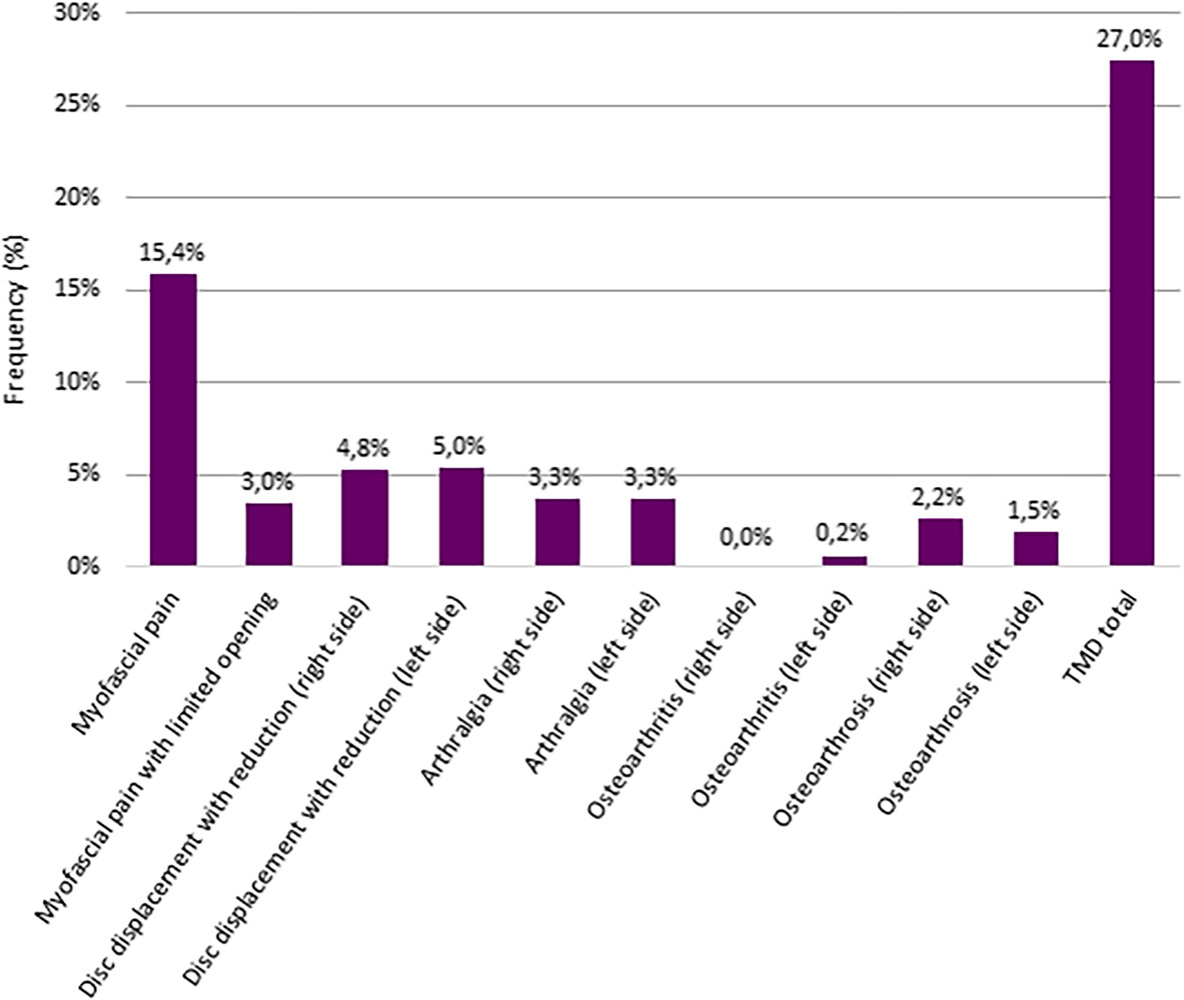
Frequencies of TMD-diagnoses in children and adolescents. Frequencies of TMD-diagnoses according to the RDC/TMD classification among 456 children and adolescents among the general population in Jeddah, Saudi Arabia

The prevalence of the children with and without TMD-diagnoses are presented in Table 2. Almost one third, 27.2 %, of the children were diagnosed with at least one TMD-diagnosis. Myofascial pain was the most the common diagnosis, followed by disc displacement with reduction, arthralgia, myofascial pain with limited mouth opening, and osteoarthrosis. One case of osteoarthritis was diagnosed, but no case with disc displacement without reduction (Fig. 2).

**Table 2:** TMD diagnoses (prevalence), TMD pain presence (S-TMD pain), duration (months and prevalence for different durations), frequency (prevalence) and intensity (0–10; current and worst as well as average during the last 6 months), headache and oral parafunctions in 456 children/adolescents from a general population in Jeddah, Saudi Arabia. Figures show frequencies (%) unless other is stated

	TMD						Non-TMD
	All	Myofascial Pain		Arthralgia	OA	DDWR	
	*n* = 124	Ia. *n* = 71	Ib. *n* = 14	*n* = 22	*n* = 12	*n* = 32	*n* = 332
Diagnoses							
*Age-group 10–13*	46.8	50.7	42.9	45.4	66.7	31.2	53.3
*Age-group 14–18*	53.2	49.3	57.1	54.6	33.3	68.8	46.7
TMD pain							
*Presence*	**71.8**	**93.0**	**100**	**59.1**	25.0	46.9	17.5
*Duration (mean (SD))*	14.5 (12.6)	15.1 (12.9)	18.1 (13)	13.4 (11.2)	10.0 (7.2)	12.5 (13.4)	12.0 (12.5)
≤1 month	7.4	10.3	0	9.1	0	0	25.5
2–6 months	27.2	19.0	21.4	31.8	33.3	53.1	27.5
>6 months	65.4	70.7	78.6	59.1	66.7	46.9	47.0
*Frequency*							
Recurrent	86.1	88.8	85.7	61.5	100	85.7	82.5
Persistent	8.1	5.6	14.3	23.1	0	14.3	0
One time	5.8	5.6	0	15.4	0	0	17.5
*Intensity (median (IQR))*							
Current	2.0 (5.0)	1.0 (5.0)	3.0 (6.0)	5.0 (4.0)	3.0 (9.0)	2.0 (5.0)	0 (5.0)
Worst	7.0 (3.0)	7.0 (3.5)	8.0 (1.0)	8.0 (2.0)	8.0 (4.0)	7.0 (4.0)	5.0 (6.0)
Average	5.0 (3.0)	5.0 (3.0)	7.0 (3.0)	5.0 (3.0)	8.0 (4.0)	5.0 (4.0)	3.0 (5.0)
Headache	**39.8**	**53.5**	**50.0**	19.0	16.7	**35.5**	8.2
Oral parafunctions							
*Clenching (daytime)*	**16.1**	**22.5**	**21.4**	9.1	0	3.1	9.0
*Grinding (nighttime)*	3.	1.4	7.14	**13.6**	0	**12.5**	2.4
*Other* ^a^	49.2	50.7	64.3	54.6	33.3	46.9	44.0

*Ia*. Myofascial pain, *Ib.* Myofascial pain with limited opening, *DDWR* Disc displacement with reduction

*S-TMD-pain* Positive answer on any of the 2 validated questions; 1) “Do you have pain in the temple, face, temporomandibular joint, or jaws once a week or more?” 2) “Do you have pain when you open your mouth wide or chew once a week or more?” [2, 16]

*SD* Standard deviation, *IQR* Interquartile range (the 75th percentile minus the 25th percentile)

^a^ Other = Mouth breathing, thumb sucking, nail biting, or tongue thrusting

The bold figures denote significant differences to the Non-TMD group (Chi-two test, *p* < 0.05)

There were no children at the age of 10 that had a TMD diagnosis, i.e. the youngest children having a TMD diagnosis were 11 years old. There were no significant differences in the prevalence of the TMD diagnoses between the two age groups (10–13 years and 14–18 years), except for disc displacement with reduction that was more prevalent in children of the older age-group (*p* = 0.017). Only girls (*n* = 14) were diagnosed with myofascial pain with limited opening (*p* < 0.001), but otherwise no sex differences were found in the prevelance of any TMD diagnoses.

### Self-reported outcomes

The frequencies of pain characteristics, such as duration, frequency and intensity, as retrieved from the RDC/TMD questionnaire, as well as of self-reported variables, such as S-TMD-pain, headache, and oral parafunctions, retrieved from the interview, for children with or without TMD diagnoses are shown in Table 2. Almost 30 % of the participants had at least one TMD-diagnosis. The TMD group reported higher frequencies of S-TMD-pain (*p* < 0.001), headache (*p* < 0.001), and daytime tooth clenching (*p* = 0.031) compared to the non-TMD group. More than 75 % of the children with TMD had never sought any physician or dentist for their pain.

The S-TMD-pain questions identified 93 of 107 children (87 %) with a TMD-pain diagnosis (myofascial pain and arthralgia). There were no significant associations between any of the TMD-diagnoses and sex, age, caries, gingivitis, nationality, economic situation, or most of the oral parafunctions (such as mouth breathing, thumb sucking, nail/object biting, or tongue thrusting). The univariate analyses showed that children diagnosed with myofascial pain reported presence of S-TMD-pain (*p* < 0.001), headache (*p* < 0.001) and clenching (*p* < 0.001) more often compared to children without TMD (*p* < 0.006). Children diagnosed with arthralgia more often reported presence of S-TMD-pain (*p* < 0.001), and grinding (*p* < 0.001), while children diagnosed with disc displacement with reduction more often reported headache (*p* = 0.004) and grinding (*p* < 0.001) than children without TMD.

In the multivariate analysis the association between a TMD-diagnosis and clenching, remained significant (Table 3). Also the associations between myofascial pain and clenching, arthralgia and grinding, and disc displacement with reduction and grinding as well as older age (14–18 years) remained significant (*p* < 0.05) (Table 3).

**Table 3:** Associations between TMD-diagnoses and sex, age daytime clenching and grinding as well as parental income in 124 children with a TMD diagnosis in a general population in Jeddah, Saudi Arabia. Figures represent odds ratios (OR) and 95 % confidence interval (95 % CI)

	TMD total		Myofascial pain		Arthralgia		Osteoarthrosis		DDWR	
	OR	95 % CI	OR	95 % CI	OR	95 % CI	OR	95 % CI	OR	95 % CI
Sex										
Male	1.0		1.0		1.0		1.0		1.0	
Female	1.3	0.9–2.1	1.5	0.9–2.5	0.9	0.4–2.3	1.0	0.2–3.4	1.2	0.6–2-8
Age										
10–13 years	1.0		1.0		1.0		1.0		1.0	
14–18 years	1.2	0.8–1.9	1.1	0.6–1.8	1.3	0.6–3.8	0.3	0.1–1.4	**2.7**	**1.2–6.3**
Clenching	**2.1**	**1.1–3.8**	**3.3**	**1.7–6.3**	0.8	0.2–3.7			0.2	0.0–1.8
Grinding	1.3	0.4–4.6	0.9	0.2–4.1	**9.0**	**2.1–38.0**			**8.8**	**2.3–33.6**
Income										
Below average	1.0		1.0		1.0		1.0		1.0	
Average	1.2	0.7–2.0	1.0	0.5–1.8	0.4	0.1–1.3	2.3	0.5–10.3	2.4	0.9–6.2
Above average	1.6	0.8–3.3	2.0	0.9–4.2	0.6	0.1–2.4	2.0	0.2–22.0	1.8	0.5–6.3

*DDWR* Disc displacement with reduction

The bold figures denote significant associations (*p* < 0.05)

### Physical functioning

Table 4 shows the physical functioning for the TMD and non-TMD groups. Children with a TMD-diagnosis scored higher on the GCPS than children in the non-TMD group (*p* < 0.05). With respect to JDL, limitations in yawning (*p* = 0.010), smiling/laughing (*p* = 0.001) and “having usual facial appearance” (*p* = 0.008) were more prevalent in children having TMD.

**Table 4:** Frequency (%) of limitations in physical function assessed with the Graded Chronic Pain Scale (GCPS) and jaw function assessed with the Jaw Disability Checklist (JDL) as well as mean (SD) maximum voluntary mouth opening (MVO) in 124 children with a TMD diagnosis and 332 children without a TMD-diagnosis from a general population in Jeddah, Saudi Arabia

	TMD total	Myofascial pain		Arthralgia	Osteoarthrosis	DDWR	Non-TMD
	*n* = 124	Ia. *n* = 71	Ib. *n* = 14	*n* = 22	*n* = 12	*n* = 32	*n* = 332
GCPS							
Grade 0	34.7	14.1	7.1	50	75	56.3	85.8
Grade 1	29.8	46.5	21.4	9.1	8.3	18.8	9.0
Grade 2	29.0	29.6	71.4	36.4	8.3	18.8	3.6
Grade 3	5.7	8.5	0	4.6	8.3	3.1	1.5
Grade 4	0.8	1.4	0	0	0	3.1	0
Jaw disability (JDL)							
Chewing	42	**41**	**57**	38	33	22	27
Drinking	2.4	1.5	7	7.7	0	6	7
Exercising	33	38	14	23	**100**	12	27
Yawning	**38**	**41**	**28**	**69**	22	22	18
Eating hard foods	61	64	50	69	25	31	45
Eating soft foods	6.8	4.6	14	7.7	0	6	9
Swallowing	10	11	7	15	0	3	5
Smiling/laughing	**46**	**51**	**36**	31	17	25	20
Talking	24	23	28	23	**25**	**25**	16
Cleaning teeth/face	**21**	**23**	**21**	31	8	12	9
Having usual facial appearance	**53**	**44**	**36**	38	22	22	69
MVO (mm)							
With pain	49.7 (7.4)	50.3 (7.7)	**42.0 (7.2)**	52.0 (5.5)	49.6 (5.7)	50.7 (6.3)	49.7 (6.1)
Without pain	43.0 (8.0)	44.8 (6.0)	**29.0 (6.0)**	44.5 (9.2)	42.5 (7.0)	43.4 (9.2)	44.2 (7.4)

*Ia* Myofascial pain, *Ib* Myofascial pain with limited opening, *DDWR* Disc displacement with reduction

*SD* Standard deviation

The results for GCPS and JDL are presented for the Non-TMD group since all children completed the RDC/TMD questionnaire before the clinical examination and diagnostics

The bold figures denote significant differences compared to the Non-TMD group? (*p* < 0.05)

Children with myofascial pain reported limitations in chewing (*p* = 0.024), yawning (*p* = 0.015), smiling/laughing (*p* = 0.001), cleaning teeth/face (*p* = 0.033) and “having usual facial appearance” (*p* = 0.001) more often than children without myofascial pain. Similarly, children with arthralgia reported limited yawning (*p* = 0.002) more frequently than children without arthralgia, children with osteoarthrosis reported limitations in exercising (*p* = 0.009) and talking (*p* = 0.049) more frequent than children without osteoarthrosis, and finally children with disc displacement with reduction reported limitations in talking (*p* = 0.001) more often than children without disc displacement.

The MVO with and without pain was within the normal range and almost identical in all groups except for in children with myofascial pain with limited opening who had significantly smaller MVO (*p* < 0.001).

## Discussion

To our knowledge, this is the first epidemiological study presenting the prevalence of TMD-diagnoses according to the RDC/TMD classification among children and adolescents younger than 14 years of age. Although several epidemiological studies have been carried out regarding TMD-signs and symptoms among adults in the general population [24] the number of structural epidemiological studies describing the prevalence of TMD-diagnoses is relatively low [25]. Even fewer studies have been carried out using the RDC/TMD protocol to investigate the TMD prevalence in children and adolescents [10, 15, 22, 26]. All but one of these studies reported signs and symptoms of TMD and did not classify their diagnoses. However, one study presented prevalences of TMD-diagnoses, but in an older population (14–25 years) [22].

In general, this study shows that about one third of Saudi Arabian children among the general population had at least one TMD-diagnosis according to the RDC/TMD criteria; myofascial pain being the most prevalent diagnosis found in approximately 15 % of the participants. In this respect, previous studies in Saudi Arabia reporting the prevalence of TMD-signs and symptoms among children and adolescents were 20–34 %, which is similar to in the current study [9, 14]. A study among Japanese children reported TMD-signs and symptoms in 23 % [27]. Other studies presented slightly lower self-reported TMD-pain prevalence, 4.2 and 22.5 % [2, 13]. In Mexico, a study reported a higher prevalence of TMD-diagnoses (46.1 %) among adolescents and young adults [22]. Furthermore, the pattern of distribution of the diagnoses in the current study seems to propose that myofascial pain and disc displacement with reduction are the most frequent diagnoses. In the study by Casanova-Rosado and co-workers [22], disc displacement with reduction showed the highest prevalence (15.6 %), followed by myofascial pain (10.9 %) and disc displacements (6.1 %). Many studies in adult populations showed similar results [28]. These findings could propose that TMD conditions in pre-pubertal age mainly are of muscular origin and then with age are complemented with intra-capsular disorders. Consequently, only one osteoarthritis case was found in this study. This is in agreement with a study indicating that arthritis seems to be uncommon among children and adolescents in Saudi Arabia [29]. On the other hand, it has been reported that 70 % of affected TMJs in children with juvenile idiopathic arthritis were clinically symptoms-free [30], indicating that there might be an underestimation of the prevalence of TMJ arthritis. Interestingly, disc displacement without reduction was not diagnosed in any child, which could be expected as it is uncommon among children and adolescents [25].

The current study showed no sex differences in the prevalence of TMD-diagnoses except for myofascial pain with limited mouth opening that was significantly more common among girls. A higher frequency of myofascial pain in girls has been previously reported [10], but in this study this was only found in the sub-group with limited opening, for which there is no clear explanation based on the results from the present study. There are, though, some other studies that have reported only small differences between boys and girls with regards to TMD-signs and symptoms [4, 5]. However, this finding is partly in contrast to recent studies in Sweden and China showing that self-reported TMD-pain (S- TMD-pain) is significantly correlated with increases in TMD prevalence both with age and among girls [2, 31]. These results are further in contrast to a study by Nilsson and co-workers (2005) showing that orofacial pain, i.e. TMD pain, is more prevalent in adolescent girls than boys [32], and to a study by LeResche and co-workers [33], which reported that female sex is a potent predictor of onset of TMD-pain. One possible reason for the lack of sex differences could be related to a higher drop-out rate of boys than girls in the present study, which could indicate a sampling bias. Another possible reason could be the lower than normal degree of physical activity in the boys group according the Social Competence scales of the Youth Self-report reported in another paper from our group [34]. Several studies have shown that there is an association between physical inactivity and musculoskeletal pain conditions [35–38], and that individuals who are physically active are significantly less likely to develop musculoskeletal pain than those who are physically inactive in long-term follow up (11 years) [39]. These results are in contrast to a previous study, also from Saudi Arabia, that reported that boys are more physically active than girls [40].

Further, this study showed that TMD is present among children above 10 years of age (i.e. as early as at 11 years of age), but that more than half of the children in the TMD group were above 14 years of age (i.e. in the age-group 14–18 years). This is in concordance with a previous study by Nilsson and co-workers (2005) which reported that TMD started to increase at the age of 12 and peaked at the age of 16, whereas Karibe and co-workers (2012) reported that more than one third of the TMD cases were even older, i.e. between 16 and 18 years [2, 41]. With the results from the present study as well as the other studies indicating that TMD onset in general occur after the age of 12, one can assume that puberty and hormonal changes might explain the higher prevalence of TMD in the older age-group (14–18) [33, 42].

In comparison to Nilsson co-workers (2005) who reported that 66.6 % of TMD-pain cases had not received treatment the current study showed that about 82 % of the TMD cases never had visited a physician or dentist for their pain [2]. The higher prevalence presented in the present study could be explained by several factors. One important factor could be the difficulties for the children and their parents to know where to go, i.e. if they should consult a physician or a dentist; there is also a lack of orofacial pain specialists in Saudi Arabia. Another reason could be the low degree of knowledge regarding orofacial pain and TMD among Saudi Arabian caregivers [43].

Previous studies have shown that headache is commonly associated with TMD in children, [12], especially with myofascial pain [44]. The significant correlation between self-reported headache and TMD in the current study supports this and also previous studies have shown that headache is common among children and adolescent in Saudi Arabia [45]. In regards to self-reported parafunctions, the current study found that clenching was statistically associated with myofascial pain, whereas, grinding was correlated with arthralgia and disc displacement with reduction. Correspondingly, previous studies found that bruxism in childhood is not only associated to TMD, but also is a potent predictor to TMD problems in adolescents and its incidence also associated with clicking in children [46, 47]. Moreover, the current result shows that one third of children with TMD reported no pain-related disability and that the majority of them had low disability and low pain intensity (Grade 1, 2). Only 6 % of the TMD cases reported high disability and high pain intensity (Grade 3, 4). One study among adult TMD patients reported that the majority of TMD patients had Grade 1 & 2, 25 % had Grade 3 & 4, but only 8 % had grade 0 (no disability) [48]. Possible explanations to this difference between children and adults could be that the orofacial muscles of young individuals have higher physiological adaptive ability during growth and development, which in turn could minimize TMD-symptoms, meaning that children get used to pain and consider it a normal feeling [49].

A previous study reported a variation of MVO between 35.5 and 43.5 mm among Saudi Arabian adolescents [50], and between 47.4 and 50.7 mm in adults, [51] whereas a study among German children reported a range between 42 and 65 mm. [52] In this study the pain-free MVO varied between 42.5 and 44.8 mm, and the MVO with pain between 49.6 and 52.1 mm, except for in children with myofascial pain with limited opening who showed significantly lower MVO. This could be expected as reduced MVO is a requirement for this diagnosis. The myofascial pain group also reported more jaw disability than the other groups and an association between jaw disability and chewing, yawning, smiling/laughing as well as cleaning the teeth/face.

A strength with the present study is the randomization of the sample participants, which permits the generalization of the results to the population of Saudi Arabia. Another strength is that the same examiner performed the clinical examinations of all children. The examiner was trained by a TMD specialist calibrated in RDC/TMD examination to a gold standard examiner, which is an additional strength. However, since more girls than boys volunteered to participate in the study, one may consider the unequal proportion between sexes as a limitation. Another limitation is that back-ground factors, such as oral parafunctions were based on self-report may not be reliable as the child may not have understood the question. To assure that the child had understood the question, the examiner also interviewed the child and explained what she meant. Still, this information should be interpreted with caution at least among the younger children.

## Conclusion

In conclusion, TMD were common among children and adolescents in Saudi Arabia (27.2 %). Self-reported orofacial pain and headache as well as bruxism were associated with a TMD-pain diagnosis and disc displacement. The low percentage of children and adolescents that sought treatment by a dentist/physician for their pains warrants the need for future research and education.

## Abbreviations

DP: Disability points
GCPS: Graded Chronic Pain Scale
RDC/TMD: Research Diagnostic Criteria for Temporomandibular disorders
SCL-90-R: Symptom Checklist-90-Revised
TMD: Temporomandibular disorders
TMJ: Temporomandibular joint
YSR: Youth Self Report Scale
